# Placebo-controlled trials in pediatrics and the child’s best interest

**DOI:** 10.1186/s13052-015-0118-6

**Published:** 2015-02-15

**Authors:** Maria Luisa Di Pietro, Renato Cutrera, Adele Anna Teleman, Maria Luisa Barbaccia

**Affiliations:** Institute of Public Health, School of Medicine “A. Gemelli”, Catholic University of Sacred Heart, Largo Francesco Vito 1, Rome, 00168 Italy; Ethics Committee, Bambino Gesù Children’s Research Hospital, Rome, Italy; Respiratory Unit, Pediatric Department, Bambino Gesù Children’s Research Hospital, Rome, Italy; Department of Systems Medicine, University of Rome Tor Vergata, Rome, Italy

**Keywords:** Clinical trials, Placebo, Minors/Parental consent, Ethics committees/consultation

## Abstract

For too long children have received medicines not sufficiently studied for their needs and, in fact, being considered as small replicas of adults, it was deemed sufficient to adjust the dosage of a drug approved for adults. Together with the limited availability of appropriate drug formulations, especially for neonates and toddlers, this approach has caused increased iatrogenic risk and/or suboptimal adherence to treatment. With the aim of encouraging the development of more efficacious and safer medicines for children, the Regulatory Agencies in Europe and U.S.A. commendably issued directives to promote adequate and well controlled pediatric clinical trials. In compliance with the agenda of the Pediatric Regulation, in the past decade the number of pediatric patients enrolled in double-blind randomized clinical trials (RCTs) is markedly increased. In order to establish the efficacy of new medicines, RCTs frequently include a placebo-control group that carries the burden of additional, and to some extent underestimated, ethical concerns with respect to trials in adults. Six years into the Pediatric Regulation implementation, off-patent drugs, most of which at present are extensively used off-label, are underrepresented in ongoing/proposed pediatric RCTs. We debate this *status quo* to assess what might be the child’s best interest. In fact, we argue that well-designed studies, in which efficacy and safety of new drugs are compared to off-patent drugs that are currently prescribed off-label, would achieve the aim of the Pediatric Regulation better and more ethically than placebo controlled RCTs.

## Introduction

Until the end of the last century children were precluded from entering clinical trials, a resolution intended to shield particularly vulnerable individuals from the unanticipated risks inherent to experimental drug exposure. Consequently, most of the drugs used everyday in children have been studied only in adults, a situation that has prompted an extensive, virtually “regular”, off-label use of drugs in pediatrics. The latter has generated a greater risk of sub-optimal treatment efficacy or of adverse effects, often caused by dosage errors due to the use of drug formulations unsuitable for a specific patient’s age, frequently leading to poor therapeutic adherence [1,2].

Finally, it has been acknowledged not only that children are exposed to a greater iatrogenic risk compared to adults, but also that they have a right to receive well studied treatments for their medical needs. Accordingly, following a similar approach taken by the FDA in the USA, at the end of 2006 the European Commission in concert with the European Medicine Agency commendably issued the Pediatric Regulation [3]. The goal of the Regulation is to encourage high quality research on drug effects in children through adequate and well controlled pediatric clinical trials, with the aim of introducing more efficacious and safer medicines into pediatric clinical practice.

The core instrument of the Pediatric Regulation is the obligation for the *Marketing Authorization Applicant* (MAA) of any new drug in development or for the *Marketing Authorization Holder* (MAH) of a drug still on-patent at the moment of the directive coming into effect, to submit a *Pediatric Investigation Plan* (PIP). This should be done as soon as possible after having acquired sufficient knowledge about efficacy and safety in adult patients.

The Pediatric Regulation provides incentives for the MAA/MAH that present data from pediatric studies, deemed of sufficient quality to include an indication for this population in the labeling of the drug. Total or partial waivers, if the drug is of no direct/potential benefit for pediatric patients as a whole or for specific age subsets, and deferrals if the PIP implementation should impose delays in the timetable of drug development/approval for adult use, can also be granted.

In the years 2007–2012, a major result of this agenda was an increased number of pediatric patients enrolled in clinical trials. Of approximately 600 PIPs submitted, about ¾ were related to not yet authorized medicines and the remainder to new indications for on-patent or off-patent medicines. In the same time period, 41 drugs received central authorization, most of which (34/41) had a mixed (pediatric/adult) indication, suggesting that their development was primarily triggered by adult medical conditions, and 34 patent-protected drugs had extended or amended indications to include the pediatric population [4].

Another measure endorsed by the Pediatric Regulation is the possibility for MAHs of any off-patent-drug of pediatric interest to apply for a *Pediatric Use Marketing Authorization* (PUMA). This procedure entails the submission of a PIP to investigate efficacy, safety and bioequivalence of appropriate formulations for children of specific age groups, in order to propose evidence-based pediatric indications.

Notably, a PUMA application is on a voluntary basis and, therefore, strongly subjected to the MAH’s economic considerations, i.e. the balance between the projected costs for the required studies and the profits that a pediatric indication may add to the existing market of the drug. EMA and its Pediatric Committee (PDCO) compile a periodically revised priority list including off-patent drugs of interest in order to answer children’s therapeutic needs in defined medical areas. Eighteen projects on off-patent drugs, presently at various levels of PIP approval/implementation, have been funded by the European Commission through the EU Framework Programme– Health, between 2007 and 2012. As of December 2012 only 1 PUMA had been granted by the EMA [4], a rather disappointing outcome, since many of the drugs used off-label in children are off-patent [3] and, therefore, ideal candidates for a PUMA application.

Strikingly, an analysis of pediatric clinical trials that were inspired by the USA Food and Drug Administration Modernization Act (FDAMA) and conducted between 1998 and 2006, found a great discrepancy between the pattern of drug prescription in children and the classes of drugs that obtained a pediatric indication. As a consequence, drugs that are most utilized in pediatrics were underrepresented in the studies aiming at obtaining pediatric exclusivity [5]. This kind of impasse appears mostly based on economic evaluations. In consideration of the different MAAs/MAHs involved, an appropriate regulatory framework as well as new methodological and statistical approaches should be devised in order to encourage/enable scientifically sound pediatric studies in which new drugs are compared, not just to placebo, but to suitable off-patent drugs that, though so far prescribed off-label, have demonstrated sufficient efficacy over the years.

## Review

### Strengths and weakness of placebo controls in pediatrics RCTS

Among various clinical trial designs, double-blind and placebo-controlled RCTs are held, by researchers and regulatory agencies, as the “gold standard”, i.e. the most methodologically rigorous approach in order to assess the effect size of prospective new medicines. Indeed, the comparison of an experimental drug to placebo allows to disregard as a specific drug-effect any improvement in the patient’s conditions related either to her/his expectancy to receive a treatment or to the natural course of the disease thus, presumably, avoiding approval of not efficacious medicines.

Placebo-controlled RCTs are required for the approval of new drugs in many instances in which the withdrawal or delay of an active treatment is deemed not to cause major or irreversible damage. However, the principle of clinical equipoise should be satisfied, in other words there should be true uncertainty with regard to the effect of the two treatments, i.e. new drug and placebo. The comparison towards a placebo-control group may depend on the medical condition as well as on the therapeutic drug class. For instance, in trials aiming at establishing the efficacy/safety of new antimicrobial and anticancer drugs the prospective new drug should be evaluated either in add-on or in comparison to the best existing standard of care, because withholding an effective treatment could dramatically reduce the patient’s probability of regaining health. The same reasoning should apply for new molecules belonging to chemical/therapeutic drug classes for which there is already a number of drugs that have been either approved or used off label for a long time (as in the case of drugs for hypertension, diabetes or immune-suppressants). Assessing the efficacy of psychotropic drugs in children and adolescent, however, represents a real scientific and ethic challenge. For instance, major depression shows a response rate to placebo even higher than in adults and the results of previous trials show that the effect size of the existing medications cannot be easily predicted [6]. For these reasons, the regulatory authorities require that efficacy of new prospective antidepressants be assessed in placebo-controlled RCTs. Pediatric major depression represents a condition associated with relevant co-morbidity, problems in family relationships, difficulties at school and social functioning, increased risk of recurrence and of suicide. Therefore, while the 8–10 weeks usually required to show efficacy in a placebo-controlled RCT may be deemed as a tolerable delay to start treatment in a child/adolescent with low-moderate severity depression that is drug-naïve or that has shown no response to existing medications, the comparison versus placebo would not be acceptable if randomization entails the withdrawal of a medication that, even if prescribed off-label, is of benefit to the patient.

According to the Declaration of Helsinki the use of placebo in medical research is allowed “for compelling and scientifically sound methodological reasons" [7], a wording of not clear-cut interpretation and that has not helped to dissipate the controversy around the issue of placebo in medical research [8].

Restrictions on the use of placebo had already been identified by Freedman [9]. The use of placebo is acceptable when: there is no standard treatment, standard treatment is no better than placebo, standard treatment is placebo, the net therapeutic advantage of standard treatment has been called into question by new evidence or effective treatment exists but is not available due to high cost or short supply.

The use of placebo is not acceptable if established known data on the drug is not taken into account (unnecessary repetition of testing), the study deprives patients of a treatment proven efficacious- thus exposing them to the risk of aggravating a morbid condition- the study entails the interruption of a treatment that is already effective and accepted by the patient, the method of administration of the therapy is invasive or causes the patient unnecessary discomfort (hospitalization, invasive diagnostic tests, stress, etc.).

In order to minimize the risks in placebo-controlled RCTs, some rules regarding experimental studies have been determined: the exclusion of patients who have an increased risk of harm (more severe disease) in case of non-response; limitation of the period of exposure to placebo to the minimum required for scientific validity; careful monitoring of the enrolled subjects; administration of the necessary treatments in the presence of severe symptoms; indication of specific criteria for the suspension of the trial in patients showing adverse events [10].

Hence, the use of placebo would be acceptable in anticipation of minimal damage and for reasons that are methodologically valid. However, the Council of Europe, taking a position on biomedical research involving persons unable to give their consent (including minors), has stated that “Research may be undertaken only if the results of the research have the potential to produce real or direct benefits to his/her health” (Chapter V, art. 15, ì) [11]. Such a statement appears at odds with the design of most placebo-controlled RCTs in pediatrics because real or direct benefits cannot be predicted for patients randomized to receive placebo, unless avoiding the potential side effects of the experimental drug can be considered a real or direct benefit. The latter, though, can hardly be included among the expected results of a RCT.

Particularly when the expected effect size is small or not easily predictable from previous studies, as it often occurs i.e. with antidepressant drugs, a placebo-controlled RCT has the advantage, *versus* an active drug-controlled RCT, of needing a lower number of patients per group in order to demonstrate a statistically significant superior efficacy of the active treatment [12]. Of course, the clinical relevance of a statistically demonstrated superior efficacy versus placebo heavily depends on the primary end-points of the study, as well as on the long-term effects which may not be evaluated in RCTs of short duration. Nevertheless, there may be several advantages associated with enrolling a lower number of patients in placebo-controlled pediatric RCTs, for instance: a) the study’s feasibility may be greater, due to the limited number of pediatric patients affected by specific diseases, and particularly when there is a need to acquire data on pharmacokinetics, efficacy and safety stratified by age groups, b) results on efficacy are obtained with a lower number of particularly vulnerable patients exposed to the unpredicted adverse effects of the new drug.

On the whole, a placebo-controlled RCT would be more time- and cost-effective. A better time-effectiveness in reaching positive results would mean a more rapid approval that would be certainly favorable for the drug manufacturer, but also for patients, because of a shorter delay in the availability of efficacious new drugs. Such a benefit should be complemented by an adequate assessment of the drug’s safety that, however, may be hindered by the short duration of the trial, based on the principle that patients should be exposed to placebo for the minimum time required to demonstrate efficacy.

A placebo control group is frequently claimed as necessary and therefore justified, on the ground that there are no standard (i.e., approved) treatments for a particular disease. Such a claim should not be so readily accepted, or at least should be analyzed case by case, when reviewing pediatric RCTs, since a placebo-controlled design would be unacceptable if there is an efficacious, though off-label, treatment in place and the study protocol entails switching the patients to placebo.

Children are daily treated with off-label drugs and as recently stated by the American Academy of Pediatrics: “… the term “off-label” does not imply an improper, illegal, contraindicated or investigational use…rather it means that the evidence required by law to allow inclusion in the label has not been approved by the FDA (or other Regulatory Agencies)…in no way does a lack of labeling signify that therapy is unsupported by clinical experience or data in children” [13].

Hence, it does not seem correct that drugs that have been used off label for years, and are deemed of sufficient efficacy/safety - though on the basis of evidence not as strong as that required for approval - to be reimbursed or paid for by National Health Systems, may not be considered as suitable comparators in pediatric RCTs that would address superiority, non-inferiority or equivalence of the prospective new drug, as applicable to the particular medical condition and drugs examined. Evidently, keeping in mind the child’s best interest, it seems that a major question to be answered concerns the definition of “standard treatment” and, thus, the minimum level of acceptable scientific evidence that, although variable for different drug classes and disease conditions, would be needed for an off label drug to be considered a proper standard treatment.

It is acknowledged, however, that with regard to international and multicenter pediatric RCTs this approach would require an effort to harmonize the heterogeneous off-label drug utilization in children of different regions (i.e. USA versus EU and among EU Nations).

### Scientific rigor, placebo and the patient’s well-being

Also in the context of a clinical trial, the primary justification for a medical act is the search of the patient’s well-being. For this reason, the evaluation of the benefit/risk ratio is fundamental when ideating an experimental project.

When determining the risks and disadvantages of a medical act, reference is made to three criteria: 1. the probability that the patient may suffer harm; 2. the severity of the harm; 3. the acceptability of harm [14]. These criteria do not appear sufficient in order to define the “minimum” damage in the case of clinical trials on minors. For this reason, it is proposed to quantify the “minimum” damage on the basis of the risk and/or discomfort that a child might encounter in everyday life and in the minor’s specific situation or during routine examinations or psychological tests [15].

However, since it is difficult to define what risk or inconvenience can be defined “minimum” and therefore acceptable, the Swiss NEK-CNE proposes to refer to the criterion of proportionality: “Rather than the use of fixed or even quantitative standards for the measurement of risk, the NEK-CNE would welcome a situational assessment on the part of ethics committees, taking into account the context of the study and the specific characteristics of the subjects concerned. Here, ethics committees should be guided by the principle of proportionality between potential harm and benefits, which is to be understood in the sense of “reasonable” risks and burdens for the child […] A study-specific assessment of burdens makes it possible to take into consideration the child’s particular situation […] and also the type and extent of benefits anticipated (individual/social, minor/major)” [16]. Complete safety cannot, however, be granted to a children who participates in a clinical trial and there is always the risk that the social advantage may override the individual benefit.

The assessment of the proportionality in the benefit/risk ratio is even more difficult in placebo-controlled RCTs, given that placebo doesn’t present in itself any real or direct benefit, unless we consider as such: 1. the absence of risks arising from the administration of an active comparator; 2. the presence of the benefits attributable to the psychobiological reactions triggered either by the overall therapeutic context or through the activation of endogenous pathways (i.e. in pain treatment) or through the notion of being the object of care and medical attention (*therapeutic ritual*) [17].

Even assuming that the utilization of placebo reduces potential iatrogenic effects, risks could instead derive from the lack of administration of an active drug, as shown by evidence of psychological damage in patients who are obliged to leave an experimental study due to the aggravation of their clinical condition [18].

As well, if a placebo treatment does not directly cause permanent adverse reactions, one must also consider the damage brought to the patient by the omission of a potentially useful treatment or by the interruption of a previously initiated treatment.

### Placebo-controlled RCTS and informed consent

Respect of patient's autonomy means allowing the patient to choose what is most appropriate for the improvement of her/his living conditions. This requires an adequate communication process that offers all the necessary information in order to obtain the patient’s consent, with the intent of removing any impediment in the exercise of autonomy. In order for information to be complete, comprehensible and result in the expression of conscious consent, it is necessary to satisfy specific requirements regarding the information’s quality and the patient’s understanding, freedom and decision-making capacity. Research, however, suggests that the participants of RCTs do not always adequately understand the studies [19,20]. In the case of clinical trials, greater attention is necessary in order to avoid the so-called “therapeutic misconception” - that is, the belief that the treatment provided has been specifically designed to meet the patient’s needs [21]. The presence of adequate information and of the patient’s consent, however, does not make any experimentation ethically acceptable, nor does it diminish the doctor’s responsibility.

The consent forms usually contain information on the study’s design, but not regarding placebo. While risks and benefits of the new treatment are listed, indications of the risks/benefits deriving from placebo are lacking [22,23]. On the other hand, it could not be otherwise, in fact, a placebo lacks a specific effect, even though it may determine a placebo-effect [24]. Given that each placebo-controlled RCTs should consider as outcomes the modification of all parameters, that is not only the physiological but also the psychological ones, the evaluation of the placebo-effect would be neither easy to obtain nor would it be reproducible. It is necessary to explain to the patient also the “nocebo” risk, partly resulting from the non-administration of the study treatment or an active comparator, when available.

Obtaining truly informed consent is even more complex when experimental studies involve children. In this case, it is even more necessary to carefully evaluate the contents and form of the informed consent [25]. The pediatric age range, in fact, not only is characterized by some specific clinical aspects due to the development of the organism, but also by a different dynamic in the doctor-patient relationship. In this case, the decisional subject does not coincide with the patient: the physician relates only with the parents, especially if the child cannot be involved because of the young age. While an older minor (14–18 years) who has demonstrated understanding and judgment (“mature” minor), may take part in the decisions, very different is the case of a child who is less than 9 years old [26].

The physician should, therefore, communicate with the parents and explain that: 1. the clinical trial is not a personalized form of treatment and that, instead, participation may preclude the possibility of receiving the standard or an off-label alternative treatment; 2. the subjects in the placebo group will not receive an active treatment; 3. some risks can derive from the non-administration of the standard/alternative treatment; 4. if the study will be interrupted, the child will begin immediately the appropriate treatment. And most importantly, the physician must explain exactly what a “placebo” is. This could, however, not be enough: the parents may have difficulty understanding the research protocol, or underestimate the possible risks, or be influenced in their decisions by the researchers’ requests.

### Placebo and the standard of the “child’s best interest” (CBI)

In clinical pediatrics, when the minor cannot be involved in decision-making process, the “child’s best interest” (CBI) is used as a standard. First used in the legal field, to make decisions on issues regarding the welfare of a child (divorce, separation, adoption, etc.), the CBI standard is very difficult to define. Those who support the validity of the CBI standard, emphasize the positive value of the analysis of each individual case and the opportunity to remind physicians of their responsibility in decisions concerning the health and life of young patients [27]. Those who criticize its validity, consider the CBI standard potentially self-destructive, individualistic, dangerous, vulnerable to forms of abuse and difficult to use since the child cannot be involved in the decision-making process and, therefore, cannot express any opinion on her/his own condition [28]. Indeed, as pointed out by the American Academy of Pediatrics, the real evaluation of a patient’s quality of life can only be made by the patient himself [29].

Pros and cons: there is a necessity for a reflection on the CBI standard not only to determine its correct content and method of application, but also to verify its validity and usefulness. In fact the limit of the CBI standard is, first of all, structural: it is the simple transposition of the principle of autonomy into a context where its exercise is not possible. To make the evaluation more objective, it would be necessary to isolate the child’s interests from those of the other subjects involved, for example: the regulatory agencies [10], the pharmaceutical companies [30], the physicians. Despite the external pressures, physicians cannot, however, forget their ethical, deontological and legal responsibilities towards their patients. Personal interests (i.e. publications/promotions) cannot be in conflict with the responsibility of caring for the patient: “If one has *to err,* one should *err o*n the patients’ side, i.e., preserve their welfare over the scientific rigor of the study” [10].

## Conclusions

The use of placebo in RCTs brings forth many questions. A clinical trial, although intrinsically bound to expand scientific knowledge, should be primarily designed after taking into proper account the patient’s interest and, therefore, the efficacy and safety of the new drug should be assessed in comparison to the best available treatment.

In the case of placebo controlled RCTs in pediatrics, this general rule should be more strictly observed because of the minor’s greater vulnerability and incapacity to be directly involved in the decisional process. Only if the prevalence of the interests of a third party can be excluded, the study meets the parameters of scientific and ethical conduct, the contents of the informed consensus forms are comprehensible and minor’s interests are respected, an ethics committee can eventually proceed to RCT's approval.

It is possible that, through an informed and prominently altruistic attitude, a child/parent may assent/consent to participate in a placebo-controlled RCT even in the presence of alternative off-label therapies. However, if the actual trend is not corrected by devising conditions to implement, whenever appropriate, adequately controlled studies in which new drugs are compared versus active drugs, even if routinely used off-label, the potential of the Pediatric Regulation to increase the utilization of more efficacious and safer medicines in pediatric clinical practice appears diminished, at least in the near future. Although the number of new medicines with pediatric indications is overall increased in the past years, it is unclear whether their benefit/risk profile is comparable, better or worse than that of old and off-patent drugs that are and have been used off-label, in many cases for decades.

In conclusion, it should be highlighted that, evidence on comparative effectiveness not only is primarily important to properly answer the patient’s therapeutic needs, but also leads to a more rational and ethical utilization of national welfare systems resources.
